# Splitting the BLOSUM Score into Numbers of Biological Significance

**DOI:** 10.1155/2007/31450

**Published:** 2007-06-04

**Authors:** Francesco Fabris, Andrea Sgarro, Alessandro Tossi

**Affiliations:** 1 Dipartimento di Matematica e Informatica, Università degli Studi di Trieste, via Valerio 12b, Trieste 34127, Italy; 2 Centro di Biomedicina Molecolare, AREA Science Park, Strada Statale 14, Basovizza, Trieste 34012, Italy; 3 Dipartimento di Biochimica, Biofisica, e Chimica delle Macromolecole, Università degli Studi di Trieste, via Licio Giorgieri 1, Trieste 34127, Italy

## Abstract

Mathematical tools developed in the context of Shannon information theory were used to analyze the meaning of the BLOSUM score, which was split into three components termed as the BLOSUM spectrum (or BLO*Spectrum*). These relate respectively to the sequence convergence (the stochastic similarity of the two protein sequences), to the background frequency divergence (typicality of the amino acid probability distribution in each sequence), and to the target frequency divergence (compliance of the amino acid variations between the two sequences to the protein model implicit in the BLOCKS database). This treatment sharpens the protein sequence comparison, providing a rationale for the biological significance of the obtained score, and helps to identify weakly related sequences. Moreover, the BLO*Spectrum* can guide the choice of the most appropriate scoring matrix, tailoring it to the evolutionary divergence associated with the two sequences, or indicate if a compositionally adjusted matrix could perform better.

## 

[1,2,3,4,5,6,7,8,9,10,11,12,13,14,15,16,17,18,19,20,21,22,23,24,25,26,27,28,29]

## References

[B1] Needleman SB Wunsch CD A general method applicable to the search for similarities in the amino acid sequence of two proteins Journal of Molecular Biology 1970 48 3 443 453 10.1016/0022-2836(70)90057-4 5420325

[B2] McLachlan AD Tests for comparing related amino-acid sequences. Cytochrome and cytochrome Journal of Molecular Biology 1971 61 2 409 424 10.1016/0022-2836(71)90390-1 5167087

[B3] Sankoff D Matching sequences under deletion-insertion constraints Proceedings of the National Academy of Sciences of the United States of America 1972 69 1 4 6 10.1073/pnas.69.1.4 4500555 PMC427531

[B4] Sellers PH On the theory and computation of evolutionary distances SIAM Journal on Applied Mathematics 1974 26 4 787 793 10.1137/0126070

[B5] Waterman MS Smith TF Beyer WA Some biological sequence metrics Advances in Mathematics 1976 20 3 367 387 10.1016/0001-8708(76)90202-4

[B6] Dayhoff MO Schwartz RM Orcutt BC Dayhoff MO A model of evolutionary change in proteins Atlas of Protein Sequence and Structure 1978 5 supplement 3 National Biomedical Research Foundation, Washington, DC, USA 345 352

[B7] Altschul SF Amino acid substitution matrices from an information theoretic perspective Journal of Molecular Biology 1991 219 3 555 565 10.1016/0022-2836(91)90193-A 2051488 PMC7130686

[B8] Karlin S Altschul SF Methods for assessing the statistical significance of molecular sequence features by using general scoring schemes Proceedings of the National Academy of Sciences of the United States of America 1990 87 6 2264 2268 10.1073/pnas.87.6.2264 2315319 PMC53667

[B9] Henikoff S Henikoff JG Amino acid substitution matrices from protein blocks Proceedings of the National Academy of Sciences of the United States of America 1992 89 22 10915 10919 10.1073/pnas.89.22.10915 1438297 PMC50453

[B10] Feller W An Introduction to Probability and Its Applications 1968 John Wiley & Sons, New York, NY, USA

[B11] Yu Y-K Wootton JC Altschul SF The compositional adjustment of amino acid substitution matrices Proceedings of the National Academy of Sciences of the United States of America 2003 100 26 15688 15693 10.1073/pnas.2533904100 14663142 PMC307629

[B12] Altschul SF A protein alignment scoring system sensitive at all evolutionary distances Journal of Molecular Evolution 1993 36 3 290 300 10.1007/BF00160485 8483166

[B13] States DJ Gish W Altschul SF Improved sensitivity of nucleic acid database searches using application-specific scoring matrices Methods 1991 3 1 66 70 10.1016/S1046-2023(05)80165-3

[B14] Sunyaev SR Bogopolsky GA Oleynikova NV Vlasov PK Finkelstein AV Roytberg MA From analysis of protein structural alignments toward a novel approach to align protein sequences Proteins: Structure, Function, and Bioinformatics 2004 54 3 569 582 10.1002/prot.10503 14748004

[B15] Zachariah MA Crooks GE Holbrook SR Brenner SE A generalized affine gap model significantly improves protein sequence alignment accuracy Proteins: Structure, Function, and Bioinformatics 2005 58 2 329 338 10.1002/prot.20299 15562515

[B16] Shannon CE A mathematical theory of communication—part I Bell System Technical Journal 1948 27 379 423

[B17] Shannon CE A mathematical theory of communication—part II Bell System Technical Journal 1948 27 623 656

[B18] Csiszár I Körner J Information Theory: Coding Theorems for Discrete Memoryless Systems 1981 Academic Press, New York, NY, USA

[B19] Schäffer AA Aravind L Madden TL Improving the accuracy of PSI-BLAST protein database searches with composition-based statistics and other refinements Nucleic Acids Research 2001 29 14 2994 3005 10.1093/nar/29.14.2994 11452024 PMC55814

[B20] Frommlet F Futschik A Bogdan M On the significance of sequence alignments when using multiple scoring matrices Bioinformatics 2004 20 6 881 887 10.1093/bioinformatics/btg498 14751984

[B21] Altschul SF Wootton JC Gertz EM Protein database searches using compositionally adjusted substitution matrices FEBS Journal 2005 272 20 5101 5109 10.1111/j.1742-4658.2005.04945.x 16218944 PMC1343503

[B22] Schäffer AA Wolf YI Ponting CP Koonin EV Aravind L Altschul SF IMPALA: matching a protein sequence against a collection of PSI-BLAST-constructed position-specific score matrices Bioinformatics 1999 15 12 1000 1011 10.1093/bioinformatics/15.12.1000 10745990

[B23] Rypniewski WR Perrakis A Vorgias CE Wilson KS Evolutionary divergence and conservation of trypsin Protein Engineering 1994 7 1 57 64 10.1093/protein/7.1.57 8140095

[B24] Hughes AL Evolutionary diversification of the mammalian defensins Cellular and Molecular Life Sciences 1999 56 1-2 94 103 10.1007/s000180050010 11213266 PMC11147084

[B25] Bauer F Schweimer K Klüver E Structure determination of human and murine -defensins reveals structural conservation in the absence of significant sequence similarity Protein Science 2001 10 12 2470 2479 10.1110/ps.ps.24401 11714914 PMC2374044

[B26] Tossi A Sandri L Molecular diversity in gene-encoded, cationic antimicrobial polypeptides Current Pharmaceutical Design 2002 8 9 743 761 10.2174/1381612023395475 11945169

[B27] Gennaro R Zanetti M Benincasa M Podda E Miani M Pro-rich antimicrobial peptides from animals: structure, biological functions and mechanism of action Current Pharmaceutical Design 2002 8 9 763 778 10.2174/1381612023395394 11945170

[B28] Selsted ME Novotny MJ Morris WL Tang Y-Q Smith W Cullor JS Indolicidin, a novel bactericidal tridecapeptide amide from neutrophils Journal of Biological Chemistry 1992 267 7 4292 4295 1537821

[B29] Kullback S Information Theory and Statistics 1997 Dover, Mineola, NY, USA

